# Kidney-targeted baicalin-lysozyme conjugate ameliorates renal fibrosis in rats with diabetic nephropathy induced by streptozotocin

**DOI:** 10.1186/s12882-020-01833-6

**Published:** 2020-05-12

**Authors:** Xiao-peng Zheng, Qing Nie, Jing Feng, Xiao-yan Fan, Yue-lei Jin, Guang Chen, Ji-wei Du

**Affiliations:** 1 Department of basic medical sciences, Taizhou University hospital, Taizhou University, No 1139 Shifu Road, Jiaojiang District, Taizhou, 318000 China; 2 College of Basic Medical Sciences, Jiamusi University, No 148 Xuefu Street, Jiamusi, 154007 China; 3 Weifang centers for disease control and prevention, No 4801 Huixian Road, Gaoxin Distric, Weifang, 261061 Shandong Province China; 4 Nursing department, Xiang’An Hospital, Xiamen University, Xiamen, 361005 China

**Keywords:** Kidney-targeted, Baicalin-lysozyme conjugate, Ameliorate, Renal fibrosis, Diabetic nephropathy, Streptozotocin

## Abstract

**Background:**

Diabetic nephropathy (DN) is one of the most common and serious complications of diabetes, and is the most important cause of death for diabetic patients. Baicalin (BAI) has anti-oxidative, anti-inflammatory and anti-apoptotic activities, which play a role in attenuating insulin resistance and protecting the kidney. Moreover, cell-specific targeting of renal tubular cells is an approach to enhance drug accumulation in the kidney.

**Methods:**

Forty-five Sprague-Dawley rats were divided into four groups. A diabetes model was created using streptozotocin (STZ) intraperitoneally injection. The four groups included: Control group (*n* = 10), DN (*n* = 15), BAI treatment (BAI; *n* = 10) and BAI-LZM treatment (BAI-LZM; *n* = 10) groups. In the current study, the renoprotection and anti-fibrotic effects of BAI-lysozyme (LZM) conjugate were further investigated in rats with DN induced by STZ compared with BAI treatment alone.

**Results:**

The results suggest that BAI-LZM better ameliorates renal impairment, metabolic disorder and renal fibrosis than BAI alone in rats with DN, and the potential regulatory mechanism likely involves inhibiting inflammation via the nuclear factor-κB signaling pathway, inhibiting extracellular matrix accumulation via the transforming growth factor-β/Smad3 pathway and regulating cell proliferation via the insulin-like growth factor (IGF)-1/IGF-1 receptor/p38 Mitogen-activated protein kinase (MAPK) pathway. BAI and the kidney-targeted BAI-LZM can utilize the body’s cytoprotective pathways to reactivate autophagy (as indicated by the autophagy markers mechanistic target of rapamycin and sirtuin 1 to ameliorate DN outcomes.

**Conclusions:**

Our data support the traditional use of *S. baicalensis* as an important anti-DN traditional chinese medicine (TCM), and BAI, above all BAI-LZM, is a promising source for the identification of molecules with anti-DN effects.

## Background

Diabetes mellitus (DM) is a life-long metabolic disease with high morbidity and mortality, which reduces the patients’ quality of life due to acute and chronic complications [1, 2]. Diabetic nephropathy (DN), a kind of complications of diabetes, is the most common and serious, eventually leads to end-stage renal disease (ESRD) [3]. It is the most important cause of death for diabetic patients. Since the progression of ESRD is irreversible, it is necessary to explore the pathogenesis of DN to identify effective methods of prevention and control. However, there are no effective therapies for patients with DN [4, 5].

The precise pathogenesis of DN is not yet fully understood. It is considered that inflammation, oxidative stress response and fibrosis were promoted by uncontrolled hyperglycemia. Certain factors initiate cellular signaling pathways that lead to apoptosis, accumulation of extracellular matrix (ECM) [6], thickening of glomerular and tubular basement membranes, and expansion of ECM and glomerular mesangial matrix [7], thus contributing to renal fibrosis and dysfunction [8]. Although the relative importance of each individual factor in the pathogenesis of the disease is not clear, nuclear factor (NF)-κB is the most important inflammatory factor in the pathogenesis of DN [9]. NF-κB activation is associated with inflammatory response in patients with DN, which could be a trigger for disease progression [10]. Activated NF-κB is translocated from the cytoplasm to the nucleus, and then induces the expression of its target genes, including transforming growth factor-β1 (TGF-β1), which is important pro-inflammatory cytokines in DN progression [11–14]. Moreover, TGF-β1 promoted the development of DN by regulating glomerular and tubulointerstitial fibrosis depended on phosphorylation and activation of Smad2 and Smad3, as well as the canonical signaling pathway [15, 16]. A large number of evidence indicates that the activation of the signal transduction pathway of the three important members of the MAPK family, namely p38 MAPK, JNK and ERK, is closely associated with the development of DN, particularly the p38 Mitogen-activated protein kinase (MAPK) signal transduction pathway, which is activated in DN and may promote the occurrence and development of DN by affecting the formation of ECM, apoptosis and cytokines [17].

Traditional Chinese Medicine (TCM), as an effective and safe therapeutic option, has been widely used to treat and control diabetes and its complications such as DN in numerous studies, and may provide insights into the mechanism of DN and constitute a beneficial supplement to drug therapy for DN [18–20].

*Scutellaria baicalensis* Georgi (*S. baicalensis*) has been widely used historically to treat DM [21, 22]. A main bioactive component of *S. baicalensis* named baicalin (BAI) has anti-oxidative, anti-inflammatory and anti-apoptotic activities [23–25]. In addition, it attenuates insulin resistance and diabetes-associated cognitive deficits [26]. Moreover, renal tubular can be used a cell-specific targeting to enhance drug accumulation in the kidney. To be mentioned, just low-molecular weight proteins can rapidly filtered and extensively accumulated in proximal tubular cells. Therefore, lysozyme (LZM, 14 kDa), as a specific carrier of renal tubular cells, have been extensively used for drug delivery [27, 28].

In the current study, the renoprotective and anti-fibrotic effects of BAI-LZM conjugate were further investigated in rats with DN induced by streptozotocin (STZ) compared with BAI treatment. The multi-target mechanism of BAI-LZM in vivo was also investigated, which may offer potential treatments for DN.

## Methods

### Chemicals and BAI-LZM preparation

BAI (purity, ≥95%) was purchased from Shanghai Yuanye Bio-Technology Co., Ltd. (cat no. CAS#21967–41-9). BAI was prepared in a 0.05% CMC-Na aqueous solution. LZM was purchased from Sigma-Aldrich (Merck KGaA; cat. no. L6876). BAI-LZM was designed and prepared in our laboratory. LZM was accurately weighed at 0.1001 g, and then dissolved in 5 ml borate buffer (0.1 mol/l, pH 7.99). BAI (0.0501 g), 1-(3-dimethylaminopropyl)-3-ethylcarbodiimide (EDC)·HCl (0.1000 g) and 1-hydroxybenzotriazole (HOBT; 0.0501 g) were extracted, dispersed in 2.2 ml acetonitrile, quickly stirred and uniformly mixed. The mixed liquid was added to LZM-borate buffer, quickly mixed, reacted at 0 °C for 18 h and then filtered. The filtered solution was purified by glucan gel G^− 25^ (Shanghai Fusheng Industrial Co., Ltd.) to remove the unreacted BAI. Finally, the solution was freeze-dried, and the resulting yellow powder was stored at low temperature.

### Characterization of BAI-LZM

#### Ultraviolet (UV)-visible absorption spectroscopy

LZM, BAI and BAI-LZM were dissolved in methanol to prepare a 1 mg/ml solution, which was placed in a special cuvette for UV-visible absorption spectroscopy.

#### Infrared spectrum

The combination of LZM, BAI and BAI-LZM was mixed with a KBr crystal at ratios ranging from 1:100 to 1:200, and finally pressed into a transparent sheet for infrared spectroscopy.

### Animal studies

All animal procedures were conducted in compliance with the Regulations for the Administration of Affairs Concerning Experimental Animals (1988.11.1), and humanely treated. The protocol was approved by the Institutional Animal Care and Use Committee (IACUC) of Taizhou University for the use of laboratory animals (Permit Number: 2007000542390). A total of 45 male adult SD rats (180–200 g, SPF grade) were obtained from the Laboratory Animal Center of Harbin Medical University. The rats were housed in plastic cages with wood shavings as cushions and maintained in a 12-h light/12-h dark cycle at 24 ± 1 °C and 55 ± 10% humidity. All animals had ad libitum access to tap water and a high-fat and sugar diet (HFSD). The rats were marked 7 days after acclimating to the facilities. DN was induced by feeding HFSD and administering STZ (Sigma-Aldrich; KGaA) intraperitoneally to the rats. A total of 10 rats were randomly selected and designated as the control group, and the remaining rats were intraperitoneally administered 65 mg/kg STZ in a 0.1 mol/l sodium citrate solution (pH 4.50) [29]. Diabetes was confirmed by measuring fasting blood glucose 72 h after STZ administration. Animals with a fasting blood glucose concentration > 16.7 mmol/l were considered diabetic and were selected as model rats for further experiments in our study. The diabetic rats were then further separated into DN (*n* = 15), BAI treatment (BAI; *n* = 10) and BAI-LZM treatment (BAI-LZM; *n* = 10) groups. Rats in the BAI and BAI-LZM groups were intragastrically administered 160 mg/kg/day BAI or BAI-LZM for 8 weeks. Animals were anaesthetized by isoflurane (4–5% for induction; 2–3% for maintenance, 0.6–0.8 L/min) in a mixture of 0.25% air and 0.5% O2; and their kidneys harvested. Blood was collected via intracardiac puncture and serum samples were stored at − 80 °C until used for biochemical measurement.

Attach: The procedures of making a high fat and sugar diet: (1) Smash the basic diet into powder; (2) Wash the fat in the water and cut it into small pieces. Put the pieces in a pot, add a little water, boil over high heat until lard has been rendered, turn to low heat, keep whisking, cook until the fat is browned, strain out the unmelted residue and then put the lard in a beaker; (3) Boil the eggs and yolk of eggs were separated; (4) Mix the basic diet, sugar, lard and egg yolk in the proportion of 59:20:18:3, mix them evenly, knead them into small cylinders and dry them on tin foil.

### Biochemical analysis

Blood samples were collected in every rat rat and serum was separated by centrifugation at 4 °C for 15 min at 3000 rpm. Biochemical parameters (blood urea nitrogen (BUN) and creatinine (Cr) were estimated using an automatic analyzer. At weeks 4 and 8, 24-h urine samples were collected from the animals, which had been fasting for 12 h in the metabolism cages the day before the experiment. Coomassie brilliant blue was used to determine the urine protein using a Bradford Protein Assay Kit (Jiancheng Biotech, Nanjing, China). The triglyceride (TG), the cholesterol (TC) and malondialdehyde (MDA) levels were measured in the supernatant of the kidney homogenate using kits (Jiancheng Biotech, Nanjing, China) according to the manufacturer’s instructions.

### Hydroxyproline levels assay

Kidney hydroxyproline content was measured by the alkaline hydrolysis method with a hydroxyproline detection kit (Jiancheng Biotech, Nanjing, China). Approximately 50 mg kidney tissue was mixed with HCl, and 1 ml alkaline hydrolysates were incubated at 120 °C overnight. Then, the hydrolysates were neutralized, mixed with chloramine T solution and oxidized for 20 min at room temperature. The oxidized product reacted with p-dimethylaminobenzaldehyde in an ethanol and H_2_SO_4_ solution at 60 °C for ~ 25 min, and the resulting chromophore was quantified spectrophotometrically at 550 nm according to a standard curve of known hydroxyproline concentrations.

### Histological examination of the kidney

A portion of the extracted kidney tissue was immediately fixed in PBS mixed with 4% paraformaldehyde and embedded in paraffin. The sections (4 μm in thickness) were stained with hematoxylin and eosin (H&E), periodic acid-Schiff (PAS) or Masson’s trichrome (MT) stain by standard procedures (Beijing Solarbio Science & Technology Co., Ltd.). Histological analysis was performed using a light microscope (DM4000B photomicroscope; Leica Microsystems, Inc.).

### Immunohistochemical (IHC) staining

After de-paraffinization, the sections were incubated with a 3% H_2_O_2_ solution to block endogenous peroxidases. Antigen retrieval was carried out using 0.1 M sodium citrate (pH 6.0) for 60 min. Sections were incubated with anti-α-SMA (1:100; BIOSS), anti-desmin (1:100; Abcam), anti-TGF-β1 (1:100; Santa Cruz Biotechnology, Inc.), anti-NF-κB p65 (1:100; Affinity Biosciences) or anti-SREBP-1 (1:100; Santa Cruz Biotechnology, Inc.) antibodies overnight at 4 °C, and a horseradish peroxidase-conjugated secondary antibody and diaminobenzidine substrate were added sequentially. Following hematoxylin counterstaining and dehydration, the sections were mounted and observed under a Leica DM4000B photomicroscope (Leica Microsystems, Inc.).

### PAS staining

The sections were placed on glass slides, de-paraffinized, stained with PAS and dehydrated with absolute alcohol. The stained tissue sections were observed using an optical microscope (Leica Microsystems, Inc.), and images of each section were obtained.

### MT staining

The sections were placed on glass slides, de-paraffinized and stained with MT stain for visualization of collagen fibers via light microscopy. The stained tissue sections were examined using an optical microscope (Leica Microsystems, Inc.) and images were obtained for each section.

### Immunofluorescence (IF) studies

Kidney sections were subjected to signal-direct IF staining of TGF-β1 (1:5; Santa Cruz Biotechnology, Inc.) or NF-κB p65 (1:100; Affinity Biosciences), followed by incubation with Alexa Flour 488-conjugated secondary antibodies (OriGene Technologies, Inc.). Nuclei were counterstained with Hoechst (Invitrogen; Thermo Fisher Scientific, Inc.). All sections were scanned, and images were acquired with a laser scanning confocal microscope (FV1000; Olympus Corporation).

### Western blot analysis

Protein samples were subjected to 8 or 10% SDS-PAGE. Proteins were next transferred to polyvinylidene difluoride (PVDF) membranes (EMD Millipore). After blocking in 5% nonfat dry milk, membranes were incubated with primary antibodies against Fibronetin (FN), E-cadherin, collagen I, SREBP-1 and Smad2/3 (1:500; Wanleibio Co., Ltd.); insulin-like growth factor (IGF)-1 receptor (IGF-1R) and caspase-9 (1:1000; Abcam); desmin (1:500; Abcam); phosphorylated (p)-NF-κB p65 and interleukin (IL)-6 (1:500; BIOSS); α-SMA (1:3500; BIOSS); TGF-β1 and p-Smad2/3 (1:500; Santa Cruz Biotechnology, Inc.); NF-κB p65 (1:500; Affinity Biosciences); IL-1β, p38 and p-p38 (1:500; CST Biological Reagents Co., Ltd.); caspase-3, mechanistic target of rapamycin (mTOR), Smad4, β-actin and GAPDH (1:500; CST Biological Reagents Co., Ltd.); and sirtuin 1 (SIRT1) (1:1000; ABclonal Biotech Co., Ltd.) overnight. Horseradish peroxidase-conjugated anti-rabbit immunoglobulin G (IgG) and anti-mouse IgG (1:7000; CST Biological Reagents Co., Ltd.) were used as secondary antibodies. The PVDF membranes were developed using Image-Pro Plus system (Tanon-2005Muti, Shanghai).

### Statistical analysis

Data were represented as the mean ± standard deviation and analyzed using a one-way analysis of variance or a two-tailed unpaired Student’s t test. *P*-values were adjusted for multiple comparisons using the Bonferroni correction. Analyses were performed using GraphPad Prism version 7 (GraphPad Software, Inc.). *P* < 0.05 was considered to indicate a statistically significant difference.

## Results

### Biosynthesis and identification of BAI-LZM

#### Reaction principle of BAI-LZM

**Fig. 1:**
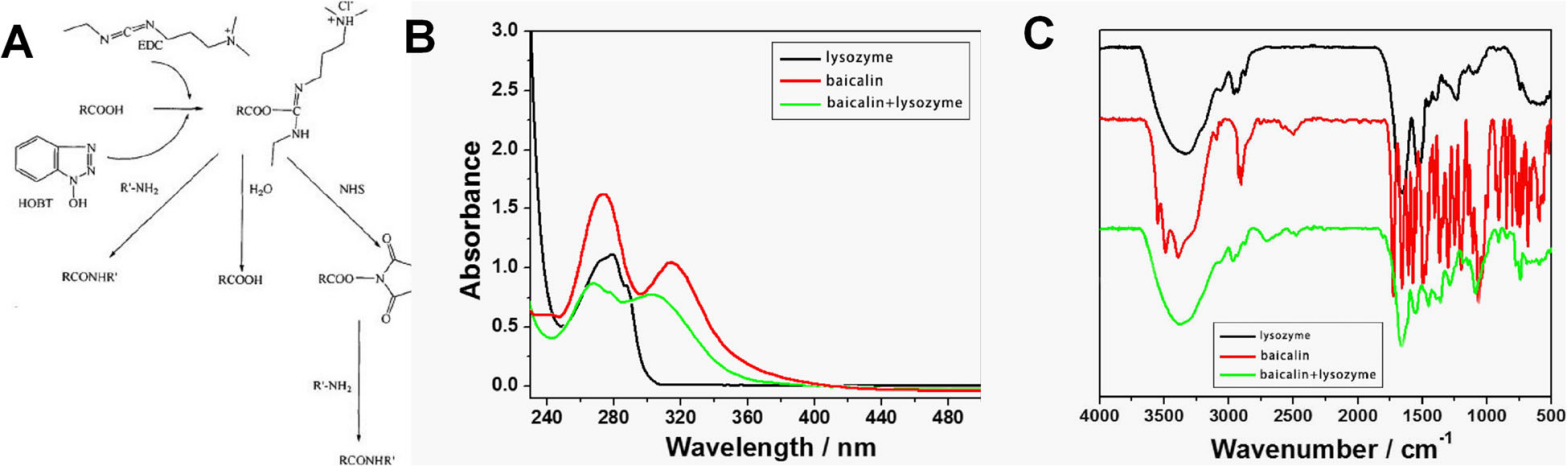
Synthesis and identification of BAI-LZM. (**a**) BAI-LZM synthesis route. (**b**) Ultraviolet-visible absorption spectrum of BAI-LZM. (**c**) Infrared spectrum of BAI-LZM. BAI-LZM, baicalin-lysozyme conjugate

The mechanism (Fig. 1a) suggested that EDC and HOBT reacted with an acid first, and then attacked with amine to produce the final product phthalamine. The results showed that it was better to incubate EDC and HOBT for a period of time before adding LZM. EDC was selected as a water-soluble dehydrating agent in the synthesis process, which satisfied he mild reaction conditions of LZM as a protein and simplified the subsequent process. HOBT was used as an auxiliary nucleophilic reagent in the synthesis process. It first reacted with a carboxylic acid in the drug molecules to produce an active lipid and then reacted with LZM, which improved the reaction rate and increased the yield.

#### UV-visible absorption spectrum of BAI-LZM

Figure 1b represents the UV-visible absorption spectrum of BAI-LZM synthesized by low temperature stirring. The methanol solution of BAI-LZM exhibited two strong absorption peaks in the region of 240–400 nm, namely at 267 and 303 nm, and the shape of the absorption peaks was approximately the same as that of BAI, which could correspond to the parent structure of flavonoids in the conjugate. However, the absorption wavelengths of the two strong peaks became shorter and blue shifted compared with BAI. This may be due to the working together of BAI and LZM influences the structural electron of BAI transfer from π to π* or n to π*. The absorption intensity of BAI at 276 nm was weaker due to the influence of the electronic transitions from π to π^*^ and from n to π^*^ in the BAI structure, which was consistent with the absorption band caused by the n-to-π^*^ transition of LZM at 277 nm. Therefore, the structure of BAI-LZM synthesized in the present study was consistent with the functional groups in the structure of BAI and LZM, and demonstrated that BAI-LZM could be prepared according to the protocol used in the present study.

#### Characterization of BAI-LZM by infrared spectroscopy

As shown in Fig. 1c, LZM had a wide and strong absorption band of -OH (polyassociated alcohol or phenol) in the range of 3500–3000 cm^− 1^, and the absorption peak near 1500 cm^− 1^ is the vibration of the benzene ring skeleton. Sharp peaks of BAI appeared in the range of 3500–3000 cm^− 1^, which was produced by the stretching vibration of the free -OH. The multi-peak near 3000 cm^− 1^ was produced by the C-H stretching vibration of the benzene ring, and the overtone peaks at 2000–1600 cm^− 1^ mainly referred to different substitution types of aromatic rings. In the present study, BAI-LZM was synthesized by chemical methods and characterized by infrared spectroscopy. The broad and strong absorption bands in the range of 3500–3000 cm^− 1^ were caused by the stretching vibration of associated alcohols or phenolic hydroxyl groups; the absorption peak at 1715 cm^− 1^ was caused by C=O stretching vibration; and the group frequency peaks near 1000 cm^− 1^ were C-H in-plane bending vibrations. By detecting and comparing the infrared spectra of BAI, LZM and the synthesized BAI-LZM, it was concluded that the structure of BAI-LZM prepared in our study was identical to the functional groups in the structure of BAI and LZM, and contained C=O, indicating that the method used in the present study was suitable to prepare BAI-LZM.

#### Kidney-targeted BAI-LZM alleviates renal impairment in rats with DN

**Fig. 2:**
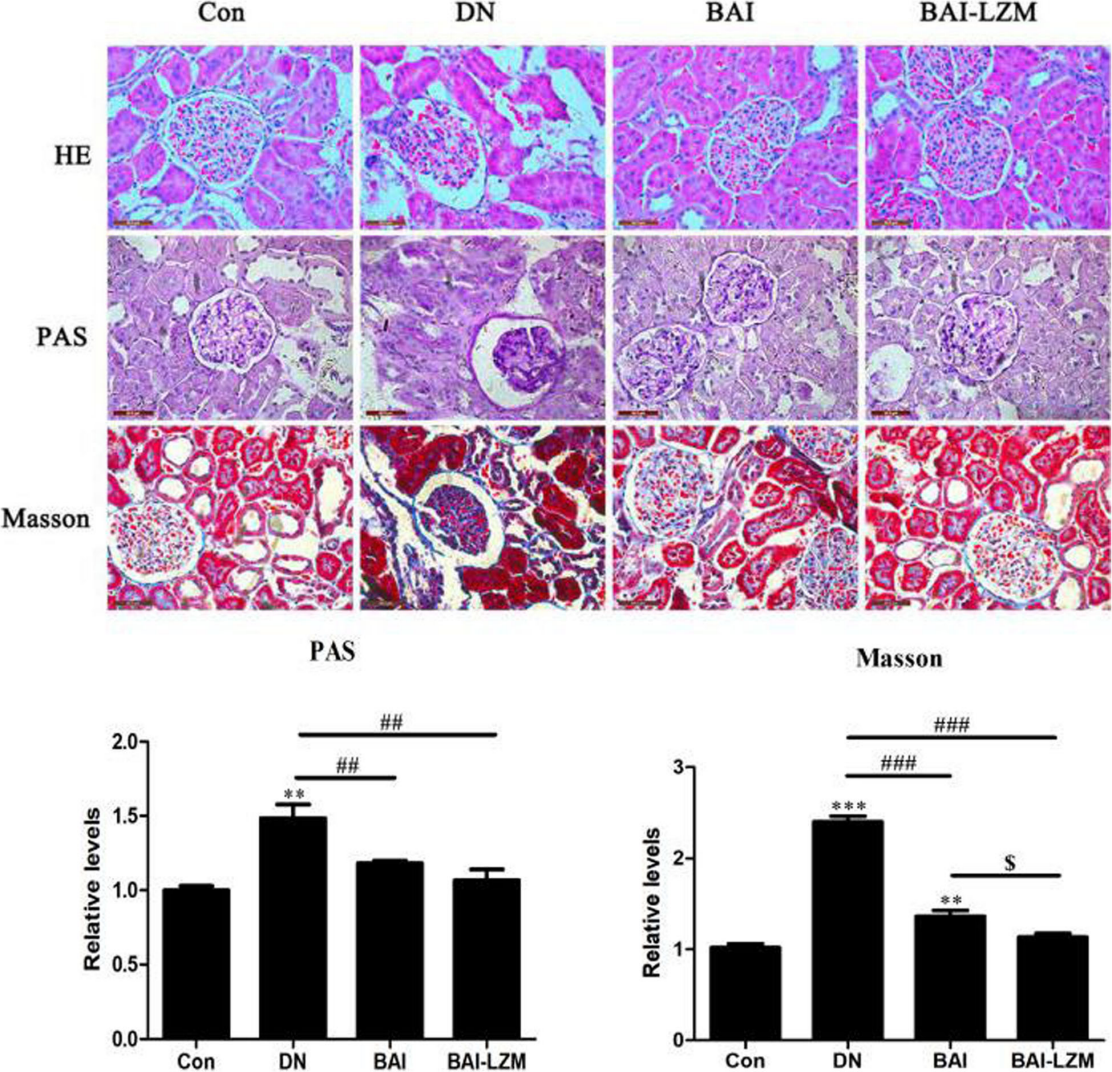
BAI-LZM alleviates renal impairment in rats with DN. Renal tissue sections from each rat were stained with (**a**) hematoxylin and eosin, (**b**) periodic acid-Schiff or (**c**) Masson’s trichrome stain. Representative images of a rat per group are displayed (magnification, × 400). Blue arrow, mesangial matrix expansion; red arrow, glomerular basement membrane thickening. Con, saline; DN, 65 mg/kg STZ; DN/B, 65 mg/kg STZ + 160 mg/kg/day BAI; DN/B + L, 65 mg/kg STZ + 160 mg/kg/day BAI-LZM. The results are representative of 3 independent experiments. Data are presented as the mean ± standard deviation. ^*^P < 0.05, ^**^P < 0.01 between the values in DN, DN/B and DN/B + L rats vs. the baseline levels (Con), as calculated by Student’s t test. ^#^*P* < 0.05, ^##^*P* < 0.01 between the DN and BAI/BAI-LZM treatment groups, as calculated by one-way analysis of variance. ^$^*P* < 0.05, ^$$^*P* < 0.01 between the BAI and BAI-LZM treatment groups, as calculated by one-way analysis of variance. *P*-values were calibrated using the Bonferroni correction. BAI-LZM, baicalin-lysozyme; DN, diabetic nephropathy; STZ, streptozotocin; Con, control

The present study further examined whether BAI and BAI-LZM improved histopathological changes or inhibited ECM accumulation and fibrosis in renal tissues. The efficacies of BAI-LZM on histopathological changes in renal tissues are shown in Fig. 2. H&E staining revealed that there were no obvious renal morphological changes in the control group, whereas significant degeneration and fibrosis were observed in the model group, and this condition was partially ameliorated by BAI and BAI-LZM treatment. Furthermore, histological evaluation revealed that diabetic kidneys exhibited increased PAS positivity in the glomerulus. By contrast, treatment with BAI and BAI-LZM reduced PAS-positive staining in the glomerulus of SD rats (*P*<0.01). MT staining showed an increase in the deposition of collagen fibrils in the glomerulus of diabetic rats. However, administration of BAI and BAI-LZM diminished the deposition of collagen fibrils in the glomerulus while reducing the intensity of staining (*P*<0.01). The effects of BAI-LZM on glomerular collagen deposition were obviously improved compared with those in the BAI treatment group (Fig. 2, *P*<0.05).

In addition, the serum Cr (Scr), urine protein and BUN contents of rats treated with BAI and BAI-LZM were further investigated to evaluate renal function. As shown in Table 1, the Scr, BUN and total urinary protein contents were notably increased in the model group compared with those in the control group, and this condition was effectively alleviated by BAI and BAI-LZM treatment. Specifically, the BAI-LZM treatment group showed better therapeutic effects compared with those caused by BAI treatment (Table 1).

**Table 1:** Effect of baicalin-lysozyme on renal function (blood urea nitrogen, serum creatinine and UPr) in rats with diabetic nephropathy

Group	N	BUN(mmol/L)	Scr(μmol/L)	UPr(mg/24h) 4 weeks	UPr(mg/24h) 8 weeks
Control	10	8.01±0.92	24.51±3.24	8.51±4.31	8.52±4.36
DN	15	16.97±3.63^**^	52.58±2.33^**^	30.98±12.66^**^	43.53±12.51^***^
DN/B	10	12.73±0.67^##^	43.77±1.73^##^	21.19±8.96^*^	19.01±9.91^##^
DN/B+L	10	10.81±0.61^##$$^	32.11±4.64^##$$^	15.66±10.36^#^	17.86±6.21^##^

Data are presented as the mean ± standard deviation ^**^P < 0.01 vs. the control group. ^#^*P* < 0.05, ^##^*P* < 0.01 vs. the DN group. ^$$^*P* < 0.01 vs. the control group

#### Effect of the kidney-targeted BAI-LZM on metabolic disorder in rats with DN

**Fig. 3:**
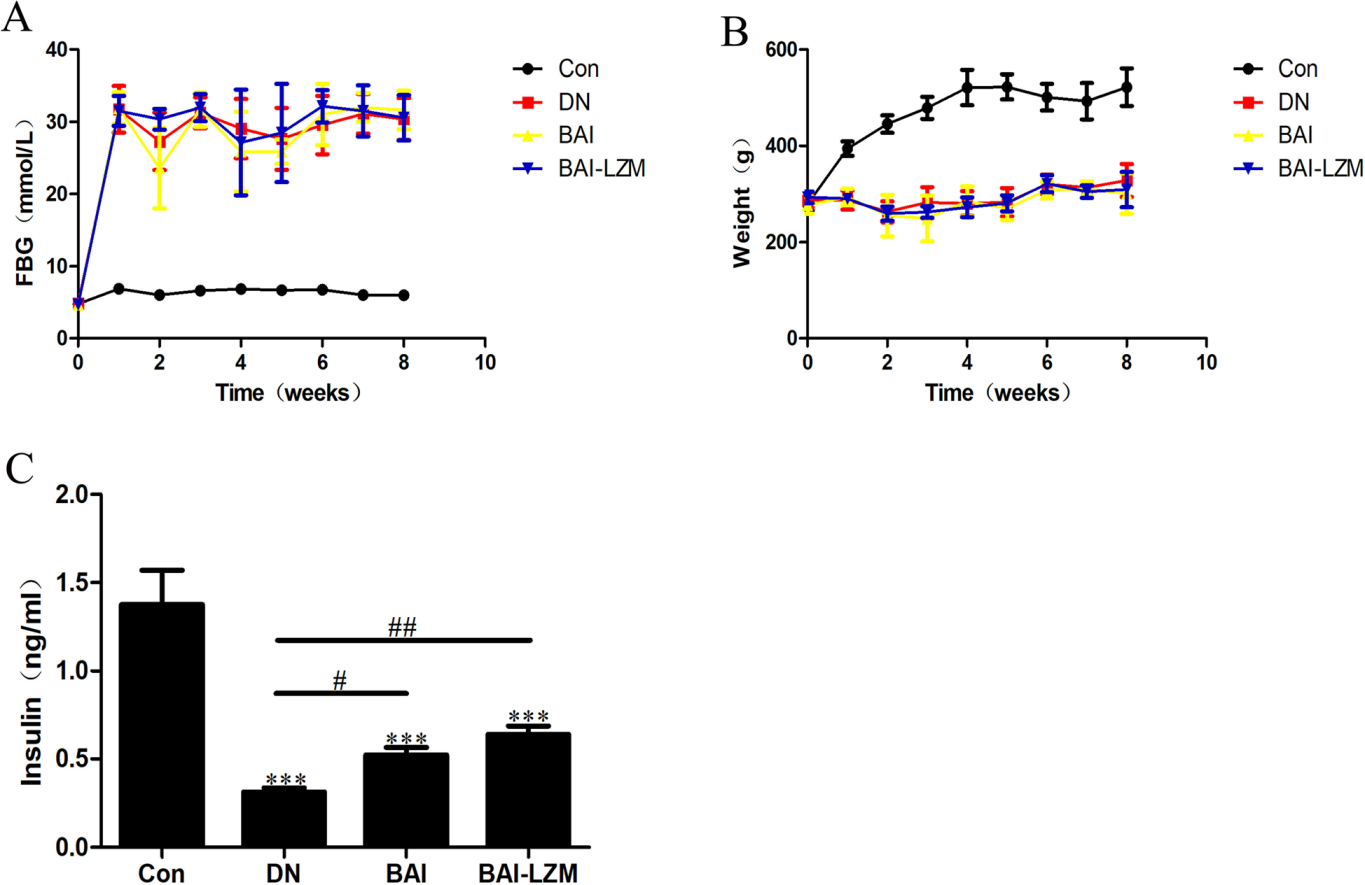
BAI-LZM improves metabolic disorder in rats with DN. (**a**) Average blood glucose level in each group. (**b**) Average body weight in each group. (**c**) Level of insulin in serum in each group. Con, saline; DN, 65 mg/kg STZ; DN/B, 65 mg/kg STZ + 160 mg/kg/day BAI, DN/B + L, 65 mg/kg STZ + 160 mg/kg/day BAI-LZM. The results are representative of 3 independent experiments. Data are presented as mean ± standard deviation. ^*^P < 0.05, ^**^P < 0.01 between the values in DN, DN/B and DN/B + L rats vs. the baseline levels (Con), as calculated by Student’s t test. ^#^*P* < 0.05, ^##^*P* < 0.01 between the DN and BAI/BAI-LZM treatment groups, as calculated by one-way analysis of variance. ^$^*P* < 0.05, ^$$^*P* < 0.01 between the BAI and BAI-LZM treatment groups, as calculated by one-way analysis of variance. *P*-values were calibrated using the Bonferroni correction. BAI-LZM, baicalin-lysozyme; DN, diabetic nephropathy; STZ, streptozotocin; Con, control

The fasting blood glucose (FBG), body weight, and insulin, TG, TC and MDA levels were further studied to reveal the effects of BAI-LZM on metabolic disorder in diabetic rats. As shown in Fig. 3, the FBG and body weight of BAI and BAI-LZM-treated rats were slightly different from those of the model group, but it was not statistically significant (P>0.05). Although the serum insulin levels in the BAI and BAI-LZM treatment groups were obviously increased compared with those in the model group (*P*<0.01), they were still below the normal levels. The levels of TC, TG and MDA were remarkably ameliorated by BAI and BAI-LZM treatment. Specifically, the BAI-LZM treatment group showed better therapeutic effects compared with those of the BAI treatment group (Table 2).

**Table 2:** Effect of baicalin-lysozyme on metabolic disorder (triglyceride, cholesterol, and malondialdehyde) in rats with diabetic nephropathy

Group	N	TG(umol/g of protein)	TC(umol/g of protein)	MDA(nmol/mg of protein)
Control	10	114.13±36.17	85.1±25.57	2.75±0.67
DN	15	1125.6±154.47^***^	1544.25±540.13^***^	12.11±2.42^***^
DN/B	10	353.42±81.87^**###^	391.24±48.03^###^	5.78±0.74^**###^
DN/B+L	10	203.68±62.34^###^	308.4±61.69^###^	3.46±0.49^###$^

Data are presented as the mean ± standard deviation ^**^P < 0.01 vs. the control group. ^#^*P* < 0.05, ^##^*P* < 0.01 vs. the DN group. ^$$^*P* < 0.01 vs. the control group

#### Kidney-targeted BAI-LZM alleviates renal fibrosis in rats with DN

**Fig. 4:**
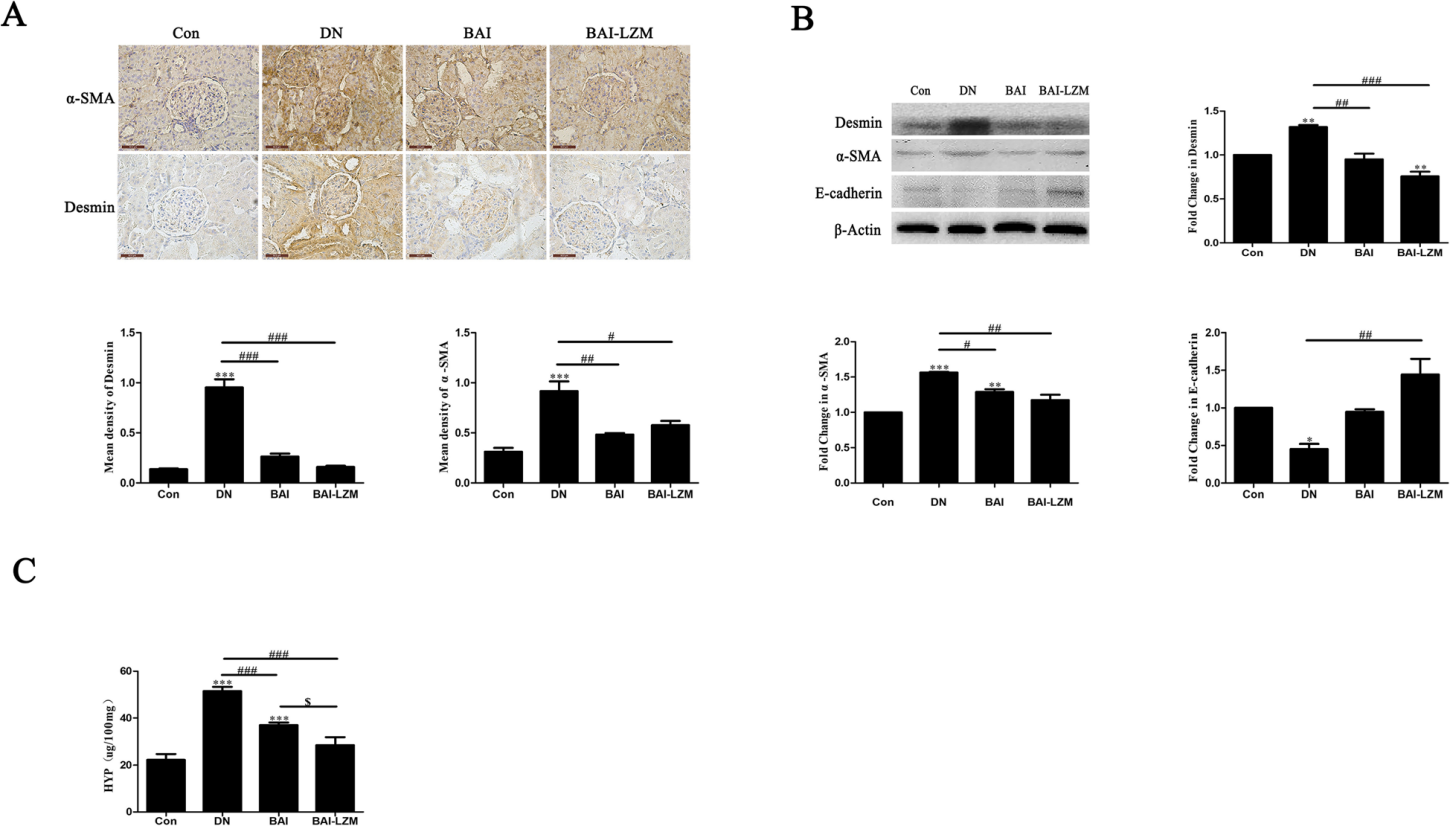
BAI-LZM alleviates renal fibrosis in rats with DN. (A) Fixed kidney tissues were stained with α-SMA and desmin, and the intensity of immunohistochemical staining for α-SMA and desmin was evaluated by optical density. Representative images of a rat per group are shown (magnification, × 400). (B) Kidney tissues were subjected to western blot assay using specific antibodies against α-SMA, desmin and E-cadherin. (C) Kidney tissues were subjected to HYP assay using a HYP detection kit. The results are representative of 3 independent experiments. Con, saline; DN, 65 mg/kg STZ; DN/B, 65 mg/kg STZ + 160 mg/kg/day BAI; DN/B + L, 65 mg/kg STZ + 160 mg/kg/day BAI-LZM. Data are presented as the mean ± standard deviation . ^*^P < 0.05, ^**^P < 0.01 between the values in DN, DN/B and DN/B + L rats vs. the baseline levels (Con), as calculated by Student’s t test. ^#^*P* < 0.05, ^##^*P* < 0.01 between the DN and BAI/BAI-LZM treatment groups, as calculated by one-way analysis of variance. ^$^*P* < 0.05, ^$$^*P* < 0.01 between the BAI and BAI-LZM treatment groups, as calculated by one-way analysis of variance. P-values were calibrated using the Bonferroni correction. BAI-LZM, baicalin-lysozyme; DN, diabetic nephropathy; STZ, streptozotocin; Con, control; HYP, hydroxyproline; α-Smooth muscle actin (α-SMA)

The endothelial-to-mesenchymal transition (EMT) is the core process of progression of renal fibrosis. The epithelial marker E-cadherin, the mesenchymal marker α-SMA and the podocyte injury marker desmin were examined to assess EMT. IHC and western blot analyses revealed that BAI and BAI-LZM treatment significantly decreased the expression levels of α-SMA and desmin (*P* < 0.001) (Fig. 4a and b), and reversed the increased expression level of E-cadherin (*P* < 0.01) (Fig. 4b) compared with rats with DN; however, it did not reach the level of the control group. In addition, hydroxyproline (HYP) were used as a measure of the collagen deposited in nephritic tissues and fibrosis. The expression level of HYP in nephritic tissues was detected by alkaline hydrolysis. As shown in Fig. 3c, BAI and BAI-LZM treatment obviously downregulated the expression of HYP in nephritic tissues compared with that in the model group (*P*<0.01). Specifically, the BAI-LZM treatment group showed better regulation effects compared with those caused by BAI treatment. These findings suggested that STZ-induced DN was accompanied by downregulation of E-cadherin and upregulation of α-SMA. Furthermore, BAI, especially BAI-LZM may partially reverse the alteration in the levels of these proteins, indicating that BAI and BAI-LZM inhibit EMT in kidney tissues.

#### Kidney-targeted BAI-LZM inhibits inflammation via the NF-κB signaling pathway in rats with DN

**Fig. 5:**
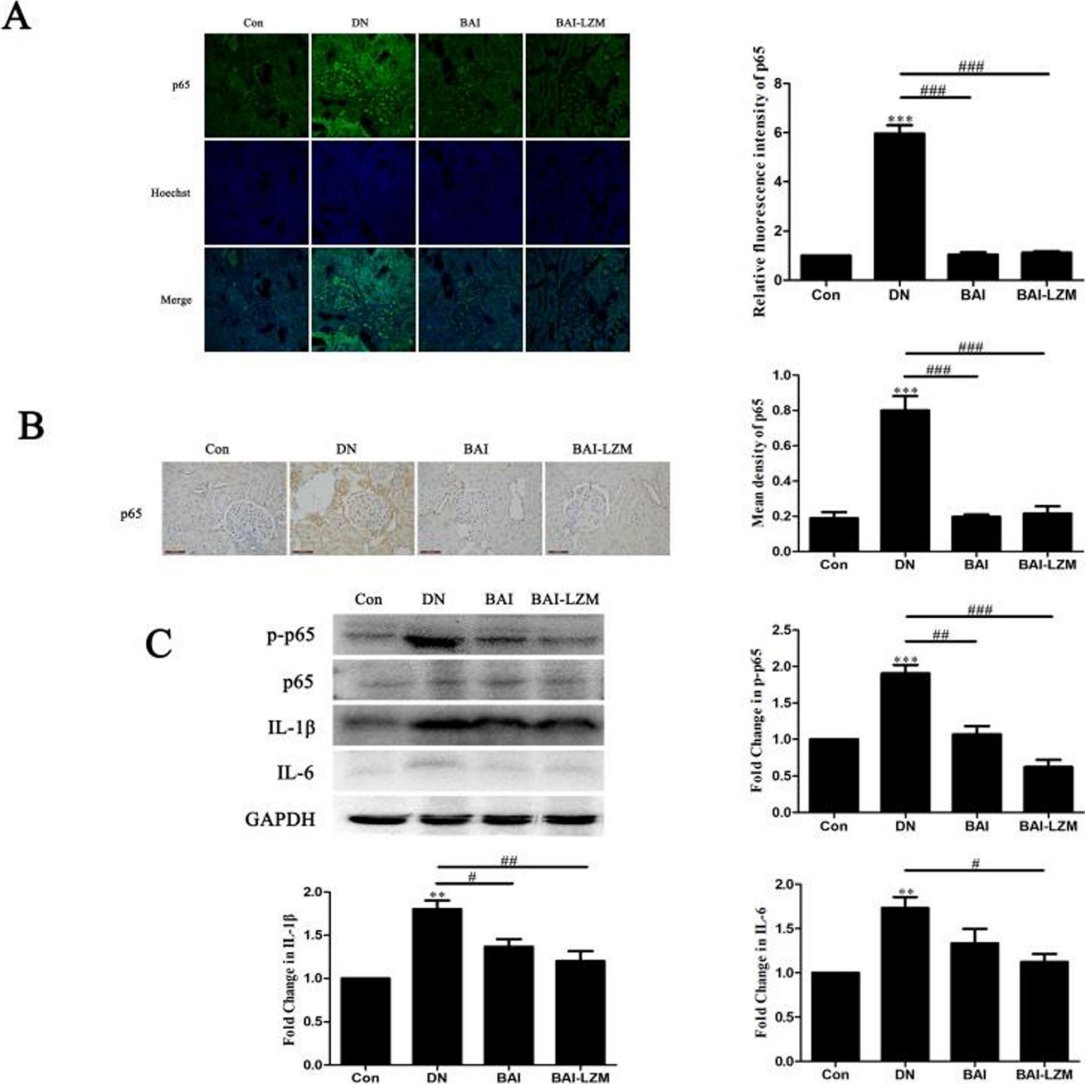
BAI-LZM inhibits inflammation via the nuclear factor-κB pathway in rats with DN. The protein levels of p65 were detected by (A) immunofluorescence and (B) immunohistochemistry (magnification, × 400). (C) The levels of phosphorylated-p65, p65, IL-1β and IL-6 protein were detected by western blotting. Con, saline; DN, 65 mg/kg STZ; DN/B, 65 mg/kg STZ + 160 mg/kg/day BAI; DN/B + L, 65 mg/kg STZ + 160 mg/kg/day BAI-LZM. The results are representative of 3 independent experiments. Data are presented as the mean ± standard deviation ^*^P < 0.05, ^**^P < 0.01 between the values in DN, DN/B and DN/B + L rats vs. the baseline levels (Con), as calculated by Student’s t test. ^#^*P* < 0.05, ^##^*P* < 0.01 between the DN and BAI/BAI-LZM treatment groups, as calculated by one-way analysis of variance. ^$^*P* < 0.05, ^$$^*P* < 0.01 between the BAI and BAI-LZM treatment groups, as calculated by one-way analysis of variance. P-values were calibrated using the Bonferroni correction. BAI-LZM, baicalin-lysozyme; DN, diabetic nephropathy; STZ, streptozotocin; Con, control; IL, interleukin

In order to determine the underlying mechanisms by which BAI-LZM inhibits renal fibrosis and decreases inflammation, the protein expression levels of NF-κB, p-NF-κB p65, IL-1β, IL-6 and mTOR in diabetic kidney tissues were assessed using IF, IHC and western blot analyses. As shown in Fig. 5a and b, the relative fluorescence intensity and expression of NF-κB p65 increased in the renal tissues of rats with DN compared with those in the control group. By contrast, BAI and BAI-LZM treatment gradually decreased the relative fluorescence intensity and expression of NF-κB p65. Additionally, the expression level of NF-κB p65 protein in the BAI group was not significantly different from that in the BAI-LZM group.

NF-κB is involved in the regulation of numerous pro-inflammatory cytokines, including IL-1β and IL-6. With the application of STZ, enhanced levels of pro-inflammatory cytokines were evident in nephritic tissues, but were reduced with BAI and BAI-LZM treatment (Fig. 5c).

#### Kidney-targeted BAI-LZM inhibits ECM accumulation via the TGF-β1/Smad3 pathway in rats with DN

**Fig. 6:**
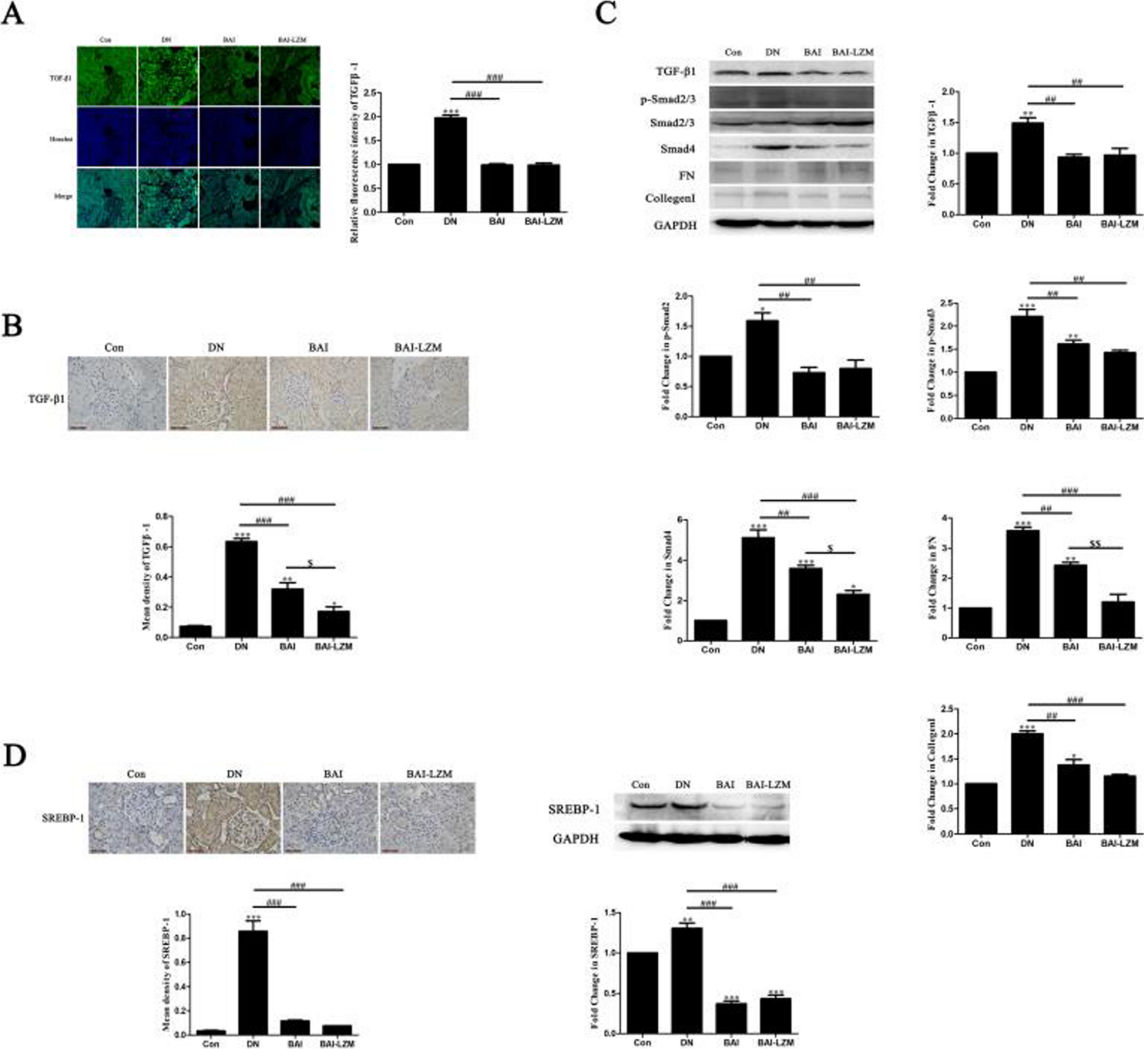
BAI-LZM inhibits extracellular matrix accumulation via the TGF-β1/Smad3 pathway in rats with DN. The protein level of TGF-β1 was detected by (A) immunofluorescence and (B) immunohistochemistry (magnification, × 400). (C) The protein levels of TGF-β1, phosphorylated-Smad2/3, Smad2/3, Smad4, FN and collagen I were detected by western blotting. The level of SREBP-1 was detected by (C) immunohistochemistry (magnification, × 400) and (D) western blotting. Con, saline; DN, 65 mg/kg STZ; DN/B, 65 mg/kg STZ + 160 mg/kg/day BAI; DN/B + L, 65 mg/kg STZ + 160 mg/kg/day BAI-LZM. The results are representative of 3 independent experiments. Data are presented as the mean ± standard deviation ^*^P < 0.05, ^**^P < 0.01 between the values in DN, DN/B and DN/B + L rats vs. the baseline levels (Con), as calculated by Student’s t test. ^#^*P* < 0.05, ^##^*P* < 0.01 between the DN and BAI/BAI-LZM treatment groups, as calculated by one-way analysis of variance. ^$^*P* < 0.05, ^$$^*P* < 0.01 between the BAI and BAI-LZM treatment groups, as calculated by one-way analysis of variance. P-values were calibrated using the Bonferroni correction. BAI-LZM, baicalin-lysozyme; DN, diabetic nephropathy; STZ, streptozotocin; Con, control; TGF, transforming growth factor

Previous studies revealed that the production of collagen, a major component of ECM, occurred in glucose-exposed renal mesangial cells, and collagen fibers were highly accumulated in the PAS-positive glomerulus of diabetic rats. IF (Fig. 6a) and IHC (Fig. 6b) analyses showed that upon application of STZ, the level of TGF-β1 was evidently high in nephritic tissues, but it was reduced with BAI and BAI-LZM treatment. Moreover, BAI-LZM treatment showed better efficacy in downregulating the expression of TGF-β1. In addition, the expression of Smad2/3/4, FN and collagen 1 (COL 1) proteins were detected in nephritic tissues of rats with DN. As shown in Fig. 6c, with the application of STZ, a reduced level of p-Smad2/3 was evident in nephritic tissues, but it increased with BAI and BAI-LZM treatment. However, the expression levels of Smad4, FN and COL 1 had the opposite trend, and with the application of STZ, the level of Smad4 protein was evident in nephritic tissue, but it was reduced with BAI and BAI-LZM treatment (Fig. 6c).

SREBPs are the most extensively studied transcription factors in lipid homeostasis, but previous studies also suggest an additional important role in matrix regulation. SREBP-1 is an important Smad3 coregulator, which can be activated by TGF-β1; interacts with Smad3 and CBP after being acetylated; and is necessary for Smad3-mediated signaling. Thus, the present study detected the expression of SREBP-1 in nephritic tissues by IHC and western blot analyses. As shown in Fig. 6d, with the application of STZ, an enhanced level of SREBP-1 was evident in nephritic tissues, but it was reduced with BAI and BAI-LZM treatment. Additionally, no significant difference was identified between the protein expression levels of SREBP-1 in the BAI group and those in the BAI-LZM group.

#### Kidney-targeted BAI-LZM regulates cell proliferation via the IGF-1/IGF-1R/p38 MAPK signaling pathway

**Fig. 7:**
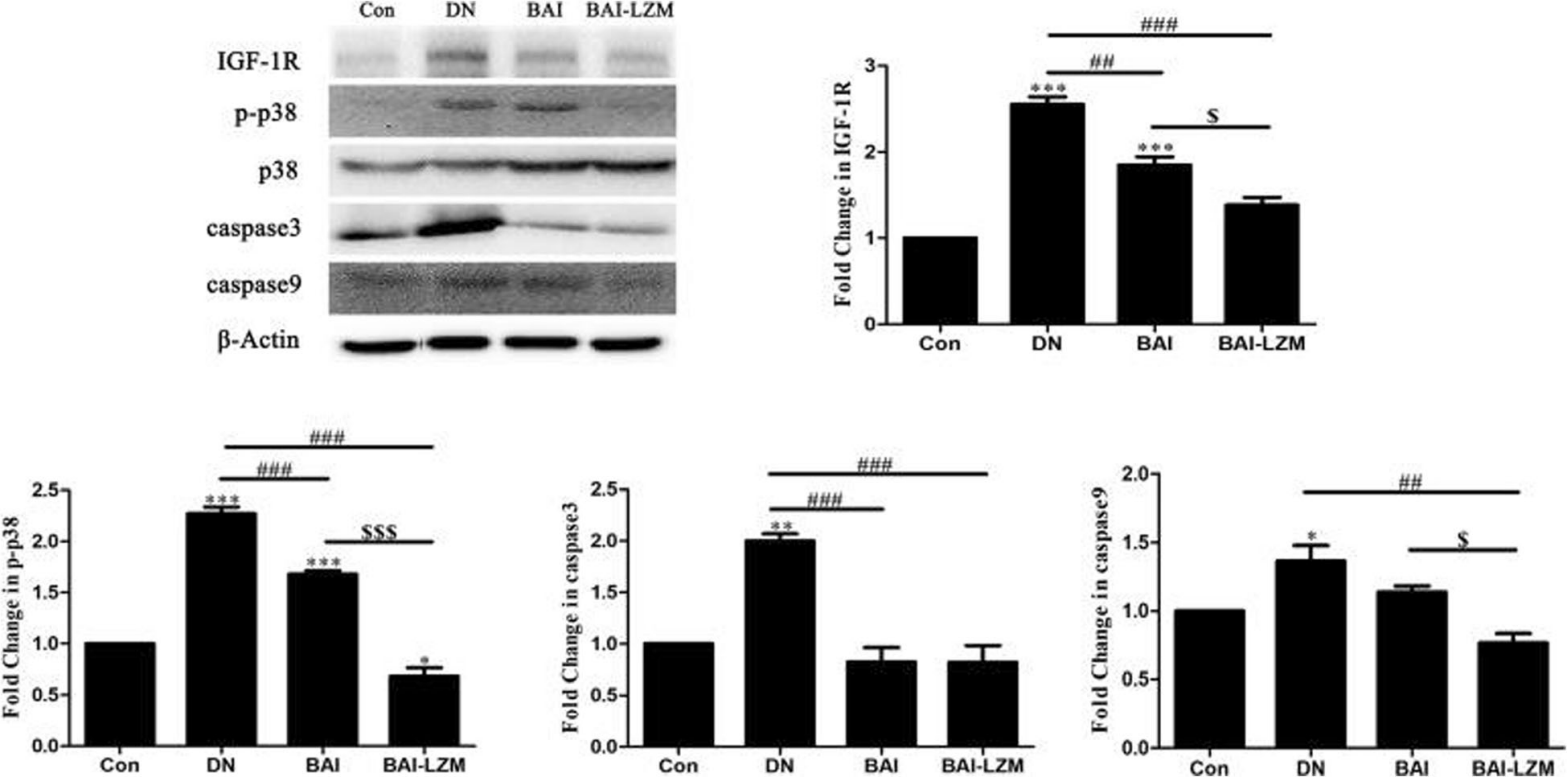
BAI-LZM regulates cell proliferation via the IGF-1/IGF-1R/p38 MAPK pathway. The protein levels of IGF-1R, phosphorylated-p38, p38, caspase-3 and caspase-9 were detected by western blotting. Con, saline; DN, 65 mg/kg STZ; DN/B, 65 mg/kg STZ + 160 mg/kg/day BAI; DN/B + L, 65 mg/kg STZ + 160 mg/kg/day BAI-LZM. The results are representative of 3 independent experiments. Data are presented as the mean ± standard deviation ^*^P < 0.05, ^**^P < 0.01 between the values in DN, DN/B and DN/B + L rats vs. the baseline levels (Con), as calculated by Student’s t test. ^#^*P* < 0.05, ^##^*P* < 0.01 between the DN and BAI/BAI-LZM treatment groups, as calculated by one-way analysis of variance. ^$^*P* < 0.05, ^$$^*P* < 0.01 between the BAI and BAI-LZM treatment groups, as calculated by one-way analysis of variance. P-values were calibrated using the Bonferroni correction. BAI-LZM, baicalin-lysozyme; DN, diabetic nephropathy; STZ, streptozotocin; Con, control; IGF-1R,; MAPK

As shown in Fig. 7, with the application of STZ, the expression of IGF-1R exhibited a rising trend in the model group compared with that in the normal control group, while BAI/BAI-LZM treatment decreased the expression of IGF-1R. In addition, enhanced levels of p-p38 MAPK, caspase-3 and caspase-9 were evident in nephritic tissues of model rats, but decreased with BAI and BAI-LZM treatment. Additionally, obvious differences were identified between the protein expression levels of p-p38 MAPK, caspase-3 and caspase-9 in the BAI group vs. the BAI-LZM group.

#### Kidney-targeted BAI-LZM plays a role in nephroprotection, which is associated with mTOR and SIRT1

**Fig. 8:**
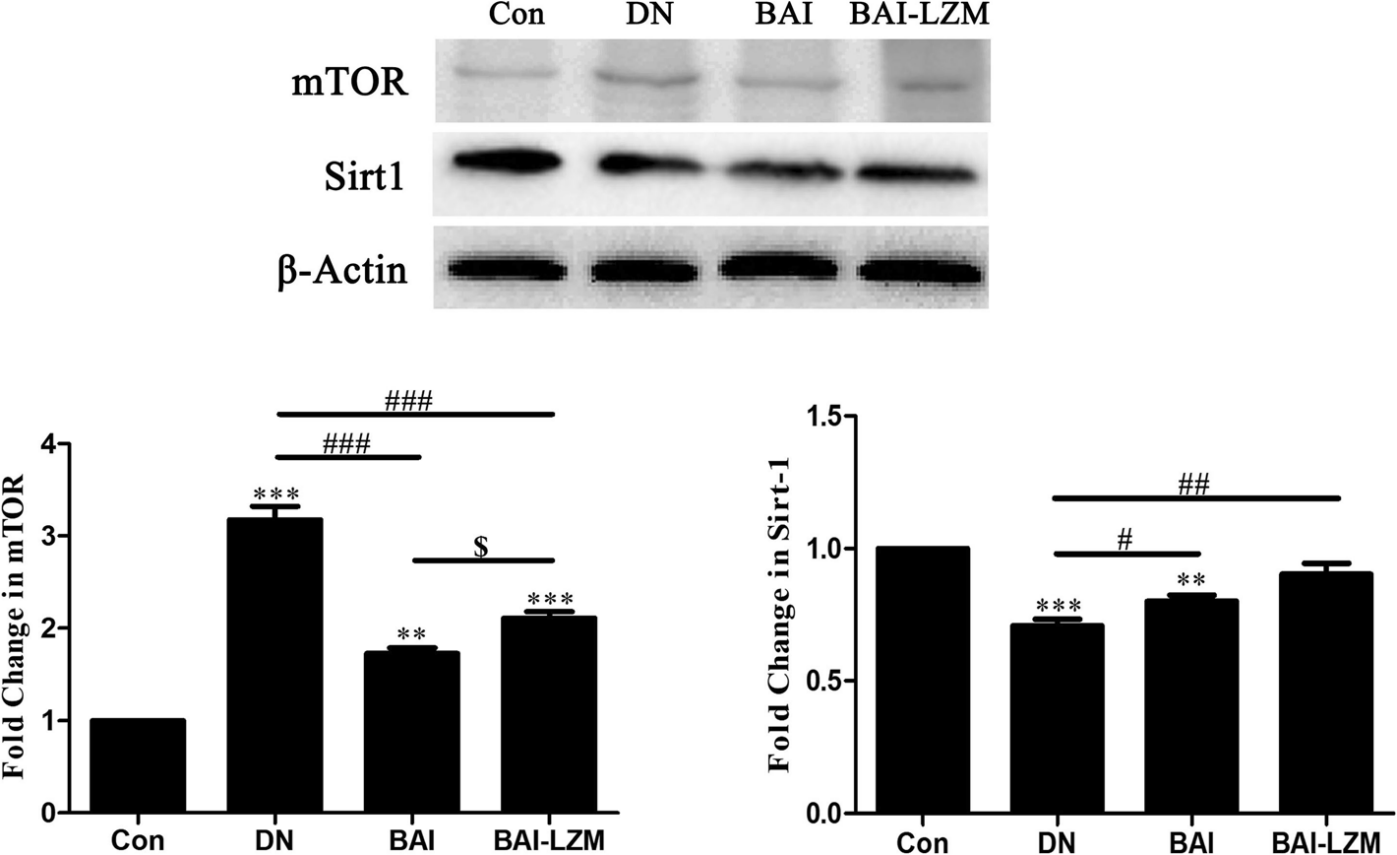
BAI-LZM plays a role in nephroprotection, which is associated with mTOR and SIRT1. The protein levels of mTOR and SIRT1 protein were detected by western blotting. Con, saline; DN, 65 mg/kg STZ; DN/B, 65 mg/kg STZ + 160 mg/kg/day BAI; DN/B + L, 65 mg/kg STZ + 160 mg/kg/day BAI-LZM. The results are representative of 3 independent experiments. Data are presented as the mean ± standard deviation ^*^P < 0.05, ^**^P < 0.01 between the values in DN, DN/B and DN/B + L rats vs. the baseline levels (Con), as calculated by Student’s t test. ^#^*P* < 0.05, ^##^*P* < 0.01 between the DN and BAI/BAI-LZM treatment groups, as calculated by one-way analysis of variance. ^$^*P* < 0.05, ^$$^*P* < 0.01 between the BAI and BAI-LZM treatment groups, as calculated by one-way analysis of variance. *P*-values were calibrated using the Bonferroni correction. BAI-LZM, baicalin-lysozyme; DN, diabetic nephropathy; STZ, streptozotocin; Con, control; SIRT1, sirtuin 1; mTOR, mechanistic target of rapamycin

Enhance the body’s cytoprotective pathways is an alternative approach for DN treatment. Thus, instead of blocking disease-driving molecules, it may be more efficient to focus on those agents that can mobilize the innate molecular defenses. Such approaches may be able to circumvent the damaging effects of glucotoxicity. As shown in Fig. 8a and b, compared with those in the DN group, with the treatment of BAI and BAI-LZM, enhanced level of SIRT1 and decreased level of mTOR were evident in nephritic tissues. Additionally, obvious differences were identified between the protein expression levels of SIRT1 and mTOR in the BAI group vs. those in the BAI-LZM group.

## Discussion

DN has always been recognized as ESRD all over the world, which may be attributed to severe hyperglycemia [30]. Researches already evaluated the various treatments and therapeutic targets, which ameliorating renal fibrosis, including drugs, endocrine hormones, complement systems and miRNAs [8, 31–33]. However, these methods have different disadvantages, which hinder the efficacy of the clinical application. Therefore, there is a pressing need for safe and efficient strategies to prevent renal fibrosis. The present study investigated the anti-fibrotic effects and potential mechanisms of BAI and BAI-LZM in a rat model of STZ-induced renal fibrosis in vivo. The results revealed that BAI and BAI-LZM could obviously ameliorate renal fibrosis, and the anti-fibrotic efficacy of BAI-LZM treatment was better than that of BAI treatment. Potential regulatory mechanisms of BAI and BAI-LZM are associated with downregulation of inflammation via the NF-κB pathway; ECM accumulation via the TGF-β1/Smad3 pathway; cell apoptosis via the IGF-1/IGF-1R/p38 MAPK pathway; and upregulation of nephroprotection via the mTOR and SIRT1 genes. Recent studies have focused on enhancing cellular defenses to treat DN as an alternative approach, which could use agents to awake the body’s cytoprotective pathways. Notably, the mTOR and SIRT1 genes are the potential targets of this intricate cellular defense. This suggests that BAI, and BAI-LZM in particular, may be a potential novel therapeutic agent for the clinical prevention of renal fibrosis.

In this study, the BAI-LZM conjugate was prepared by chemical synthesis. UV-visible absorption and infrared spectroscopy were used to identify the characteristics of the BAI-LZM conjugate. Our results showed that the structure of BAI-LZM prepared in our study was identical to the functional groups in the structure of BAI and LZM, and contained C=O, indicating that the method used was feasible to prepare BAI-LZM (Fig. 1).

At present, people changed the original concept of DN, which is a purely vascular disease, just considered that DN is a multi-dimensional, multi-cellular condition. Current treatment strategies for DN can delay but not prevent progression of disease, nor can address the marked emotional, physical and financial costs. Thus, novel therapeutic agents must be identified, specifically efficient, multi-pathway, multi-perspective anti-fibrotic agents. Multiple TCM products have been used to treat chronic kidney disease, particularly DN [34]. Single herbal TCM products, particularly monomers derived from a single herbal TCM component, present numerous advantages over conventional medical approaches for renal protection in DN due to their reduced toxicity and/or side effects [35–37]. BAI is one of the major bioactive components of *Scutellaria radix,* and has various pharmacological activities, including anti-inflammatory, anti-tumor, anti-microbial, anti-oxidant, eye protective and anti-viral properties [38, 39]. These biological activities are mainly associated with its antioxidant properties as well as its abilities to inhibit enzymes and regulate immune responses and certain pro-inflammatory mediators. In addition, BAI has certain therapeutic effects on hepatic fibrosis, cardiac fibrosis, pulmonary fibrosis and renal interstitial fibrosis [40–42]. Considerable evidence suggests that podocyte injury plays an important role in the development and progression of DN [43]. Consistently, in our animal experiments it was observed that BAI and BAI-LZM treatment obviously downregulated the expression of α-SMA, podocyte injury marker: desmin and HYP, and reversed the increased expression level of E-cadherin in nephritic tissues compared with those in the model group (*P<0*.01). Specifically, BAI-LZM treatment showed better regulatory effects than BAI treatment (Fig. 4). Relative study found that EMT, perhaps correlate with a primary underlying mechanism of ECM and fibrogenesis, because of the loss of epithelial characteristics and the acquisition of a mesenchymal phenotype, has been hypothesized to be a [44]. Our results indicated that the area of interstitial collagen deposition was reduced, and EMT in renal tissues was suppressed, certificating its protective effect on renal fibrosis.

In DN, thickening of the glomerular capillary wall, deposition of ECM, and expansion and proliferation of mesangial cells were the main reasons, which altered the renal functions [44]. These pathological changes induced the alterations of several biochemical parameters in the concentration of blood and urine [45]. In addition, the TCM prescription HLJDD (including Baicalin) possessed potent lipid-modulating effect on type 2 diabetic rats, the levels of TC and TG, were decreased [46]. In the present study, BAI and BAI-LZM markedly protected against renal damage (Fig. 2), although did not alter FBG or weight, and upregulated the level of insulin while downregulating the levels of TG, TC, MDA, BUN, Scr and albuminuria in serum/urine compared with those in the model group (Fig. 3 and Tables 1 and 2). Perhaps the main reasons which FBG or weight has been altered by BAI or BAI-LZM treatment, correlated with the level of insulin. BAI or BAI-LZM treatment increased insulin production, but didn’t recover to the normal level compared with normal control rat. If we will continue to treat diabetic rat using BAI or BAI-LZM a long time, FBG or weight should be change obviously. Specifically, the BAI-LZM treatment group showed better therapeutic effects than the BAI treatment group. The present study demonstrated that BAI, and BAI-LZM in particular, protected against renal damage and regulated several biochemical parameters to repair impaired renal function.

As the characteristic of DN, glomerular hypertrophy and accumulation of ECM can induce glomerulosclerosis, interstitial fibrosis and progressive renal insufficiency [47]. The complex interplay of hyperglycemia, mechanical stress, oxidative stress, micro-inflammation and increased expression of prosclerotic growth factors such as TGF-β and angiotensin II just triggered the occurrence of these pathological changes [6, 48]. Together, cellular signaling pathways were activated by these factors result in apoptosis and accumulation of ECM; however, we do not know the relative importance of each individual factor in the pathogenesis of the disease. In addition, it is well known that DM is a low-grade inflammatory disease, and the pathogenesis of DN is complex and involves low-grade inflammation [49]. Among the signaling pathways involved in DM, the NF-κB pathway has been extensively reported to be involved in the inflammatory response [48, 50, 51]. Looking for the novel inflammatory molecules may be in favor of the development of new therapeutic strategies. Thus, transcription factors, pro-inflammatory cytokines, chemokines, adhesion molecules, Toll-like receptors, adipokines and nuclear receptors, are total associated with inflammatory pathways in DN, just as candidate molecular targets of DN treatment. NF-κB, as a kind of the transcription factors, correlated with the pathogenesis of DN. Under basal conditions, NF-κB is not appeared in the cytoplasm by the inhibition of IκB. Upon activation by numorous factors, including ROS and MAPK, the IKK subunits, particularly IKKα/β, are phosphorylated. The p65 and p50 subunits, just as activated and phosphorylated form of the NF-κB subunits, translocate toward the nucleus, where they regulate a surplas of pro-inflammatory cytokines, such as TGF-β1, IL-6, TNF-α and IL-1β [52]. The hyper-phosphorylation of p65 and the levels of IL-1β and IL-6 upon STZ treatment confirmed the findings of previous reports that NF-κB activation associated with the occurrence of DN. However, BAI and BAI-LZM treatment obviously inhibited the levels of the pro-inflammatory cytokine in DN rat, as it could markedly suppress the critical pro-inflammatory cytokines production (IL-1β, IL-6) (Fig. 5).

During the process of renal fibrosis formation, TGF-β1, as a key pro-fibrotic regulator plays a central role. Among its three isoforms, namely TGF-β1, 2 and 3, all types of renal cells total can produced TGF-β1 [53] and acts as a pro-fibrotic regulator in several ways: i) Fibrotic proteins such as FN and collagen I can be induced by TGF-β1independently; and ii) TGF-β1 can induced the phosphorylation of Smad2 and Smad3, and then formed the oligomeric complexes includingSmad2, Smad3 and Smad4 [54]. Following, the oligomeric complexes shift to the nucleus result for the transcription of target genes, including FN, collagen I and collagen IV [55, 56]. Based on the above results, TGF-β1 and Smads could be star therapeutic targets for renal fibrosis. In addition, coupled with our previous studies, the transcription of TGF-β1 can be control by SREBP-1, which makes SREBP-1 a major regulatory factor of TGF-β1-mediated fibrotic kidney disease [57]. In the present study, STZ treatment induced TGF-β1 production and activated downstream Smad2, Smad3 and Smad4, thus increasing the expression of FN, collagen I and SREBP-1 in nephrotic tissues (Fig. 6). BAI and BAI-LZM reversed these effects, and BAI-LZM exhibited better efficacy in inhibiting ECM accumulation. We speculated that BAI, and particularly BAI-LZM, could inhibit renal fibrosis via a TGF-β/Smads-dependent signaling pathway.

IGF-1R signaling participates in the regulation of cell proliferation and apoptosis [58–60]. Accumulated data indicate that IGF-1R exerts its main action through the p38 MAPK signaling pathways [61, 62]. P38 MAPK forms part of a subfamily of the MAPK superfamily. Under the stimulation of various factors, including hyperglycemia, pro-inflammatory factors and oxidative stress, the phosphorylation of MAPK kinase and activation of p38 MAPK by inducing the phosphorylation of p38 MAPK residues [63]. Activated p38 MAPK signaling participates in the processes of cell growth, differentiation, apoptosis, environmental stress response and inflammatory responses [64]. Accumulated data showed that p38 MAPK regulates and frequently promotes renal inflammation and apoptosis [65, 66]. Moreover, the attenuation of fibrosis by BAI and baicalein was partially attributed to inhibition of fibroblast proliferation and induction of apoptosis [67]. In our study, hyper-phosphorylation of p38 and the levels of caspase-3 and caspase-9 increased upon treatment with STZ. However, BAI and BAI-LZM treatments appeared to reduce the expression of apoptotic proteins in DN, as they could markedly suppress the occurrence of apoptosis in nephrotic tissues (Fig. 7). In addition, insulin analogs promote an excess IGF-1R in STZ-induced diabetic rats, which could overstimulate the mitogenic signaling pathways [68]. We found that BAI and BAI-LZM treatment increased the level of insulin production, which indicate that BAI and BAI-LZM regulate other signaling pathway and/or cooperate with insulin regulated the activation of IGF-1/IGF-1R/p38 signal pathway. The results suggest that nephritic fibrosis in rats with DN induced by STZ was improved by BAI and BAI-LZM treatment via the p38 MAPK signaling pathway by downregulating apoptosis.

At present, DN can be treated by regulating the balance between glycaemia and hypertension. However, these standard treatment regimen in clinical just delay but not prevent disease progression [69]. Thus, an alternative approach to DN treatment could involve the use of agents to harness the body’s cytoprotective pathways. The nutrient-sensitive process of autophagy is downregulated in DN [69, 70]. Moreover, SIRT1 [71] and mTOR [72] are associated with the regulation of autophagic disorders in diabetic rats. Evidence suggests that, in DN, the occurrence of autophagy is not enough; just accelerate the process of renal fibrosis [73]. Interesting, rapamycin kinase (mTOR), the activation of autophagy-inhibiting genes, recreated the key features in animal models [74]. Moreover, levels of the mitochondrial protector SIRT1 were significantly reduced in DN [75]. As expected, our results showed that the protein expression levels of mTOR and SIRT1 in renal tissues of rats with DN were downregulated and upregulated, respectively, by BAI and BAI-LZM treatment (Fig. 8). These results suggest that BAI, and BAI-LZM in particular, can inhibit renal fibrosis via reactivating autophagy. Now, utilizing this intricate cellular defense mechanism to looking for the potential targeting appears promising. However, this therapeutic strategy is still primary; we need to identify more sophisticated approaches to reactivate autophagy.

**Fig. 9:**
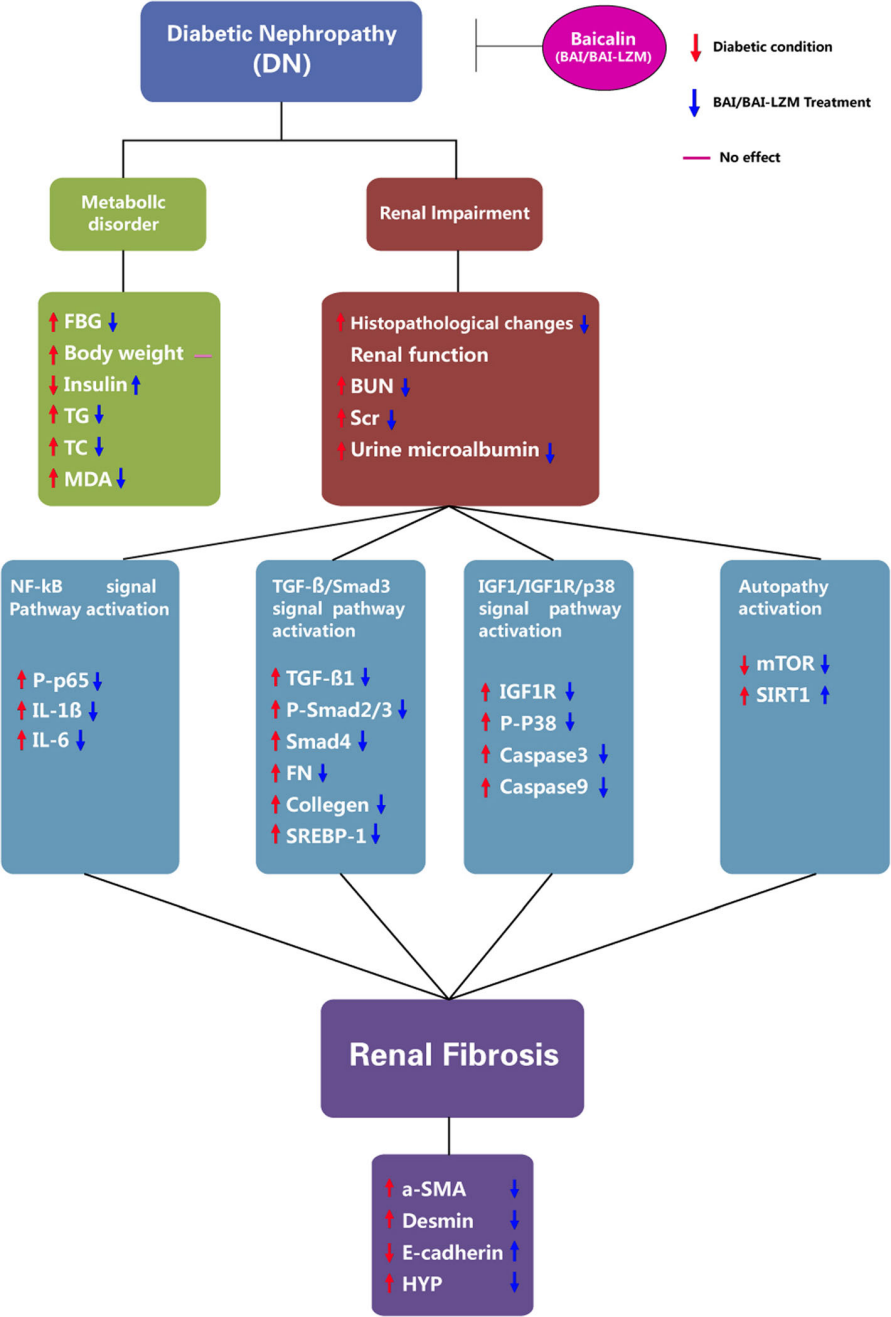
Summary of the proposed role of BAI on STZ-induced DN. BAI, and particularly the kidney-targeted BAI-LZM conjugate, produced inhibition of renal fibrosis and inflammation via the NF-κB, TGF-β1/Smad3 and IGF-1/p38 MAPK signaling pathways. BAI and BAI-LZM can harness the body’s cytoprotective pathways to reactivate autophagy (autophagy markers, mTOR and SIRT1) to ameliorate DN outcomes

In summary, our results indicate that BAI, and particularly the kidney-targeted BAI-LZM conjugate, produced inhibition of renal fibrosis and inflammation via the NF-κB, TGF-β1/Smad3 and IGF-1/p38 MAPK signaling pathways. BAI and BAI-LZM can harness the body’s cytoprotective pathways to reactivate autophagy (autophagy markers, mTOR and SIRT1) to ameliorate DN outcomes (Fig. 9). BAI and BAI-LZM attenuate structural and functional damages of the kidney in a STZ-induced DN model in vivo. Our data support the traditional use of *S. baicalensis* as an important anti-DN component in TCM, and BAI is a promising source of novel molecules with anti-DN effects.

## Conclusions

Despite the benefits derived from strict control of glucose and blood pressure, numerous patients continue to enter ESRD. Thus, develop new effective therapeutic approaches further to understand the mechanism of DN, and then prevent the progression of DN, is particularly important. Several studies have strengthened the therapeutic rationale of TCM in the treatment of DN. Compared with other anti-DN TCM products, BAI, especially BAI-LZM has more advantages for DN treatment, including multi-target, multi-dimension and multi-mode effects. To be mentioned, BAI has a absolute therapeutic efficacies, which is utilizing reactivation of autophagy, except for the traditional anti-inflammatory and anti-fibrosis. However, the feasibility and safety of these therapeutic approaches as well as the clinical applicability of TCM in human DN remain to be further investigated.

## Data Availability

The datasets analysed during the current study are available from the corresponding author on reasonable request (misschenguang75@163.com).

## Abbreviations

DM: Diabetes mellitus
DN: Diabetic nephropathy
ESRD: End-stage renal disease
BAI: Baicalin
STZ: Streptozotocin
ECM: Extracellular matrix
NF-κB: Neclear factor-κB
TGF-β1: Transforming growth factor-β1
TCM: Traditional Chinese Medicine
LZM: Lysozyme
UV: Ultraviolet
HFSD: High-fat and sugar diet
BUN: Blood urea nitrogen
Cr: Creatinine
TG: Triglyceride
TC: Cholesterol
MDA: Malondialdehyde
H&E: Hematoxylin and eosin
IHC: Immunohistochemical
PAS: Perildic acid-Schiff
MT: Masson’s trichrome
IF: Immunofluorescence
PVDF: Polyvinylidene difluoride
FN: Fibronetin
IGF-1R: Insulin-like growth factor-1 receptor
mTOR: Mechanistic target of rapamycin
SIRT1: Sirtuin 1
EMT: Endothelial-to-mesenchymal transition
HYP: Hydroxyproline
COL-1: Collagen-1
α-SMA: α-smooth muscle actin
